# Evaluation of interventions to prevent vasovagal reactions among whole blood donors: rationale and design of a large cluster randomised trial

**DOI:** 10.1186/s13063-023-07473-z

**Published:** 2023-08-10

**Authors:** Amy McMahon, Stephen Kaptoge, Matthew Walker, Susan Mehenny, Philippe T Gilchrist, Jennifer Sambrook, Naim Akhtar, Michael Sweeting, Angela M Wood, Kathleen Stirrups, Ryan Chung, Sarah Fahle, Elisha Johnson, Donna Cullen, Rosemary Godfrey, Shannon Duthie, Louise Allen, Paul Harvey, Michael Berkson, Elizabeth Allen, Nicholas A Watkins, John R Bradley, Nathalie Kingston, Gail Miflin, Jane Armitage, David J Roberts, John Danesh, Emanuele Di Angelantonio

**Affiliations:** 1 British Heart Foundation Cardiovascular Epidemiology Unit, Department of Public Health and Primary Care, University of Cambridge, Cambridge, UK; 2 Victor Phillip Dahdaleh Heart and Lung Research Institute, University of Cambridge, Cambridge, UK; 3 National Institute for Health and Care Research Blood and Transplant Research Unit in Donor Health and Behaviour, University of Cambridge, Cambridge, UK; 4 Health Data Research UK Cambridge, Wellcome Genome Campus and University of Cambridge, Cambridge, UK; 5 NHS Blood & Transplant, Blood Donation, Barnsley, UK; 6 School of Psychological Sciences, Macquarie University, Sydney, NSW Australia; 7 Centre for Emotional Health, Macquarie University, Sydney, NSW Australia; 8 Department of Haematology, University of Cambridge, Cambridge Biomedical Campus, Cambridge, UK; 9 National Institute for Health and Care Research BioResource, Cambridge University Hospitals NHS Foundation, Cambridge Biomedical Campus, Cambridge, UK; 10 NHS Blood and Transplant, Bristol, UK; 11 University of Leicester, Leicester, UK; 12 British Heart Foundation Centre of Research Excellence, University of Cambridge, Cambridge, UK; 13 Cambridge Centre of Artificial Intelligence in Medicine, University of Cambridge, Cambridge, UK; 14 NHS Blood & Transplant, John Radcliffe Hospital, Oxford, UK; 15 NHS Blood & Transplant, Ashford, UK; 16 Data, Analytics and Surveillance, UK Health Security Agency, Nobel House, London, UK; 17 MRC Population Health Research Unit, Nuffield Department of Population Health, University of Oxford, Oxford, UK; 18 Radcliffe Dept of Medicine and BRC Haematology Theme, University of Oxford, Oxford, UK; 19 Department of Human Genetics, Wellcome Sanger Institute, Hinxton, UK; 20 Health Data Science Centre, Human Technopole, Milan, 20157 Italy; 21 NHS Blood and Transplant, Cambridge, UK

**Keywords:** Vasovagal reactions, Blood donors, Blood donation, Cluster randomised trial, Cross-over, Stepped-wedge, Factorial design

## Abstract

**Background:**

Vasovagal reactions (VVRs) are the most common acute complications of blood donation. Responsible for substantial morbidity, they also reduce the likelihood of repeated donations and are disruptive and costly for blood services. Although blood establishments worldwide have adopted different strategies to prevent VVRs (including water loading and applied muscle tension [AMT]), robust evidence is limited. The Strategies to Improve Donor Experiences (STRIDES) trial aims to reliably assess the impact of four different interventions to prevent VVRs among blood donors.

**Methods:**

STRIDES is a cluster-randomised cross-over/stepped-wedge factorial trial of four interventions to reduce VVRs involving about 1.4 million whole blood donors enrolled from all 73 blood donation sites (mobile teams and donor centres) of National Health Service Blood and Transplant (NHSBT) in England. Each site (“cluster”) has been randomly allocated to receive one or more interventions during a 36-month period, using principles of cross-over, stepped-wedge and factorial trial design to assign the sequence of interventions. Each of the four interventions is compared to NHSBT’s current practices: (i) 500-ml isotonic drink before donation (*vs* current 500-ml plain water); (ii) 3-min rest on donation chair after donation (*vs* current 2 min); (iii) new modified AMT (*vs* current practice of AMT); and (iv) psychosocial intervention using preparatory materials (*vs* current practice of nothing). The primary outcome is the number of in-session VVRs with loss of consciousness (i.e. episodes involving loss of consciousness of any duration, with or without additional complications). Secondary outcomes include all in-session VVRs (i.e. with and without loss of consciousness), all delayed VVRs (i.e. those occurring after leaving the venue) and any in-session non-VVR adverse events or reactions.

**Discussion:**

The STRIDES trial should yield novel information about interventions, singly and in combination, for the prevention of VVRs, with the aim of generating policy-shaping evidence to help inform blood services to improve donor health, donor experience, and service efficiency.

**Trial registration:**

ISRCTN: 10412338. Registration date: October 24, 2019.

**Supplementary Information:**

The online version contains supplementary material available at 10.1186/s13063-023-07473-z.

## Introduction

### Background and rationale

Vasovagal reactions (VVRs), the most common acute complication related to blood donation, are responsible for substantial morbidity, especially when accompanied by complications such as fall and fractures [1–3]. VVRs also discourage individuals to donate again, reducing the likelihood of repeated donations by more than 50% [4, 5]. Furthermore, VVRs can disrupt the throughput and yield of donor sessions due to social contagion “ripple effects” on other donors attending the same sessions [6]. VVRs are not only harmful for donors but also costly and highly disruptive for blood services.

Operating at different stages of blood donation, the principal mechanisms believed to underlie donation-related VVRs include the direct effects of removal of ~500mL of blood; the stress of giving blood (e.g. fear of needles, pain, sight of blood); and orthostatic effects superimposed on a relative hypovolaemic state after donation [7, 8]. Based on this understanding, various strategies to prevent VVRs have been proposed. For example, the World Health Organisation (WHO) recommends pre-donation hydration and applied muscle tension (AMT), a technique to increase blood flow and blood pressure by tensing and releasing muscles. The EU Domaine Project, which also endorses the use of pre-donation hydration and AMT, additionally recommends caffeine loading, distraction techniques, supportive care, and educational material [9–11]. Blood services worldwide have adopted varying strategies to prevent VVRs; water loading and AMT are the most common [12–15].

A previous meta-analysis of randomised trials has, however, shown that there is limited evidence to support water loading or AMT [16–18], because previous studies have typically involved small sample sizes; non-representative populations; non-robust outcomes definitions; and sub-optimal randomisation methods. These concerns apply even more sharply to the sparser evidence available on other interventions used by some blood services, including isotonic drinks [19–21] prolongation of post-donation recovery time, and psychosocial educational materials [22, 23]. Hence, blood services need more robust evidence to help shape and inform their currently diverse approaches to prevent VVRs.

To evaluate the effects of four interventions for the prevention of VVRs among whole blood donors, we have designed the Strategies to Improve Donor Experiences (STRIDES) trial, a large cluster-randomised trial across all sites of the national blood service in England (National Health Service Blood and Transplant [NHSBT]). The trial aims to provide evidence-based policies to reduce VVRs among blood donors, leading to improvements in donor health, donor experience, and service efficiency. A subsidiary aim is to advance understanding of the determinants of VVRs and to develop prevention strategies for VVRs tailored to specific donor sub-populations.

We confirm that recruitment into the trial was ongoing at the time this manuscript was submitted for publication.

### Objectives {7}

The primary objective of STRIDES is to determine the optimum intervention(s) or combination of interventions to prevent VVRs in whole blood donors. A secondary objective is to advance understanding of the determinants of VVRs and to help develop prevention strategies for VVRs tailored to specific donor sub-populations (e.g. stratified by demographic, biological, psychosocial and other characteristics).

#### Hypothesis

We will test the hypothesis that the implementation of one or more interventions, singly or in combination, will reduce VVRs when compared to current practice in NHSBT.

### Trial design {8}

#### Feasibility study

Prior to the initiation of STRIDES, a feasibility study was conducted in five blood donation sites (comprising 3 donor centres and 2 mobile teams) over a period of 15 weeks (25 March 2019 to 26 June 2019) to establish if the protocol for a cluster-randomised trial could be implemented successfully without disrupting routine blood collections. The feasibility study was also used to assess the acceptability of the interventions by blood donors. It showed that the implementation of the four different interventions under evaluation here−either individually or in combination−was practical and did not adversely affect routine blood collection (Supplementary Fig. 1). Furthermore, feedback from donors and staff showed very good acceptability and adherence to the interventions (Supplementary Fig. 2).

#### Main trial design

STRIDES is a cluster-randomised cross-over/stepped-wedge factorial trial of four interventions to reduce VVRs involving randomisation of all 73 blood donation sites (mobile teams and donor centres) of NHSBT in England. Four interventions were compared to current practice in NHSBT (i.e. *vs* standard of care as reference): (i) 500-ml isotonic drink before donation (*vs* current 500-ml plain water); (ii) 3-min rest on donation chair after donation (*vs* current 2 min); (iii) new modified AMT (*vs* current practice of AMT); and (iv) psychosocial intervention using preparatory materials (*vs* current practice of nothing). Each blood donation site (i.e. “cluster”) has been randomly allocated to receive one or more interventions during four 9-month periods (i.e. a 36-month total period) using principles of cross-over (for isotonic drink and time spent in donation chair, to reduce between-cluster confounding and maximise statistical power), stepped-wedge (for the modified AMT and psychosocial interventions, since these interventions cannot be “un-learned” by participants once introduced to the trial) and factorial trial design to construct the temporal sequence of interventions (Supplementary Table 1). A cluster randomised trial design was preferred as it was most practicable for service-wide adoption without disrupting routine blood donation and minimised contamination across interventions.

## Methods: participants, interventions and outcomes

### Study setting {9}

**Fig. 1:**
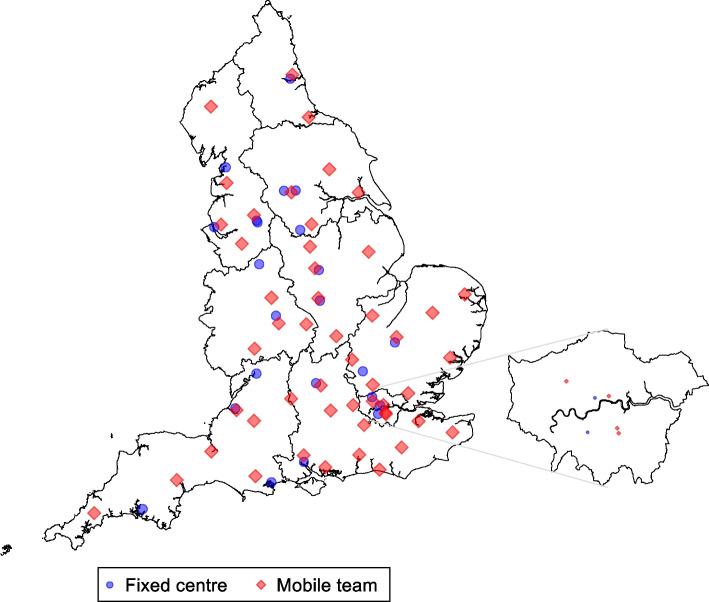
A map showing the locations of the blood donation sites. Mobile team points relate to the centre where the team is based, however, teams travel to different locations within their area for whole blood collections

NHSBT collects whole blood from donors at both static donation centres and mobile teams. Recruitment in STRIDES has been taking place in all 73 blood donation sites of NHSBT located across England (Fig. 1). The STRIDES trial has been embedded into the existing routine operational framework of NHSBT (Supplementary Fig. 3). To support additional functions required in the trial, we have established an academic trial coordinating centre at the Department of Public Health and Primary Care, University of Cambridge. In addition to supporting the trial’s core scientific activities, the coordinating centre provides a helpdesk to respond to queries from participants about the trial and maintains a study website (www.strides-study.org.uk). The academic coordinating centre has worked closely with the STRIDES administration team based within NHSBT.

### Eligibility criteria {10}

All blood donors who were eligible to donate and attended a donation site during the study period (2^nd^ of November 2019 to 3^rd^ of November 2022) were included in the trial. Eligibility criteria for donation require that donors are generally fit and well, aged over 17 and weigh between 50 and 158 kg. Donors had the opportunity to opt out of their pseudonymised data being used in the analysis. No donors were excluded from the study unless they chose to opt out.

### Who will take informed consent? {26a}

We have followed the guidance of the Ottawa Statement on the Ethical Design and Conduct of Cluster Randomized Trial [24]. Given the nature of the trial and the interventions used, the need for individual consent was waived by the ethics committee. To alert donors that the study was taking place, poster advertisements have been placed in each trial site. Furthermore, electronic messages about the trial have been sent to all donors prior to its start, plus annual reminders. By completing a simple form, donors have had the opportunity to opt out of the trial, declining the sharing by NHSBT of their pseudonymised data with the academic coordinating centre at the University of Cambridge.

To avoid biases that could undermine the interpretation of study results (e.g. greater awareness of fainting that could result in “social contagion” or other group triggers that lead to increasing fainting), we have not individually informed blood donors about the trial’s specific aim to investigate strategies to reduce VVRs [6, 25]. Instead, if donors asked about changes in donation sessions, donation staff have advised that the changes are being tested for the purposes of enhancing the donation experience (rather than explicitly focusing on VVRs). This communications approach has been consistent with that used in other service-wide changes recently made to routine blood donation practice by NHSBT.

### Additional consent provisions for collection and use of participant data and biological specimens {26b}

As noted above, the need for individual consent into the trial has been waived by the ethics committee. For the STRIDES BioResource sub-study, however, donors have needed to provide their individual and informed consent if they agree to join the NIHR BioResource and provide their surplus blood (taken from the satellite pouch of their routine donation) for research purposes. In particular, donors who have agreed to participate in the STRIDES BioResource have received NHSBT’s routine screening for donation eligibility, i.e. measurement of haemoglobin via copper sulphate test, followed by the Hemocue™ test for those that fail the initial test. Research nurses from NHSBT have, after providing an explanation of the STRIDES BioResource, sought consent from those donors invited to join the STRIDES BioResource. Donors wishing to participate have been asked to complete, with the assistance of a donor carer (if required), an electronic consent form using a tablet device. Alternatively, donors have been able to complete a paper version of the consent form, attached to the back of the Participant Information Sheet.

## Interventions

### Explanation for the choice of comparators {6b}

Interventions are tested versus current NHSBT practice (i.e. the standard of care).

### Intervention description {11a}

As noted above, our trial is evaluating different interventions: (1) 500ml of isotonic drink before donation; (2) 3-min rest on donation chair after donation; (3) modified applied muscle tension (AMT); and (4) a psychosocial intervention using preparatory materials. Table 1 summarises the interventions and key differences from current NHSBT practice. Working across three regional groups, a combination of NHSBT and University of Cambridge staff (including members of the public) have trained NHSBT donor centre staff in trial procedures. All sites began the first set of interventions on the 2^nd^ of November 2019, with changes to interventions occurring every 9 months, according to the pre-specified intervention assignments of the trial’s clusters. As the assessment of intervention effects relies on comparing event rates in periods with and without interventions applied, the 9-month period length was chosen to be long enough to accrue sufficient numbers of the primary outcome events and give contrasts that meet the statistical power requirements.

**Table 1:** Details of the proposed interventions

**Type of intervention**	**Mechanism and rationale**	**Content**	**Timing**	**Delivery**	**Key differences with current practice**
**Isotonic drink**	Suggested to prevent both immediate and late-onset VVRs due to its additional effect of decreasing relative hypovolemia and increasing orthostatic tolerance through electrolyte replacement	Isotonic drink (500 ml): ~350 mg sodium, ~0.03g sugar	Before donation	Cup given to all donors with information posters advertised (isotonic drinks prepared prior to donor arrival/tablet given to donor to add to water)	Sodium content to help restore electrolytes lost during donation
**Applied muscle tension**	Physical manoeuvres including tensing/releasing of skeletal muscles to combat orthostatic intolerance Static contractions of the skeletal muscles result in the emptying of large capacitance veins and increased venous return, blood pressure, and ultimately greater cerebral perfusion Other suggested mechanisms include anxiety reduction and distraction	Keeping legs crossed throughout exercise Tensing legs and buttocks and including the abdomen Instructions to “breathe normally” 5-s contractions and release To exercise AMT following donation chair and leaving site, if symptoms	Immediately upon arrival at donation chair	Information handout with instructions given to all donors	Keeping legs crossed throughout exercise (current practice: crossing and uncrossing) Tensing legs and buttocks and including the abdomen (current practice: no abdomen) Instructions to breathe normally (current practice: no breathing instructions) 5-s contractions and release (current practice: 10-s release) To exercise AMT following donation chair or leaving site, if symptoms (current practice; no such recommendation)
**Psychosocial**	Fear, pain, blood-related stimuli, lack of social support, and lack of a sense of control are associated with autonomic regulation, hemodynamic changes, blood pressure changes, and VVRs Preparation materials have been suggested to reduce VVRs	Providing information on the donation process, coping strategies, and various areas of possible apprehension (e.g. fear, pain, physical reactions)	Upon arrival and registration	Information handout given to all donors immediately following registration	Providing relevant information and coping strategies to blood donors directly, including related strategies such as distraction (not currently provided in usual practice)
**Chair**	Following the donation, the hypovolemic state combined with the orthostatic stress of suddenly standing leads to an increased risk of VVRs Recovery time in the donation chair in the upright position after removal of the donation needle has been suggested to attenuate orthostatic stress, reducing the amount of sudden blood pooling and consequent VVRs	Two minutes in donate/recovery position followed by 1 min in the final rest position	Immediately following needle-withdrawal	Chair inclined by staff manually	Duration of time in chair following donation (i.e. a total of 3 min vs. 2 min in current practice)

### Criteria for discontinuing or modifying allocated interventions {11b}

As this pragmatic trial was embedded into routine donation services, the study has been open to the following minor amendments. First, if a participant has not wished to receive the trial’s interventions, they have been able to receive NHSBT’s standard interventions (e.g. water loading rather than the isotonic drink evaluated in the trial). Second, the trial has been conducted during the COVID-19 pandemic, meaning that during the study NHSBT has routinely implemented service-wide revisions with the aim of enhancing donor and staff safety at donation sessions, including limiting the number of donors per session and removing hot drinks previously offered after donations.

### Strategies to improve adherence to interventions {11c}

Using a commercially available feedback device (https://www.feedbacknow.com/), we assessed participants’ adherence to the trial interventions during the feasibility study. Overall, adherence to the new interventions was very similar to that observed with standard NHSBT practice. During the 3-year trial period, we have assessed adherence to interventions by random site visits. Research nurses at NHSBT have visited each blood collection team at least once during each intervention arm, using a protocol to check and record that trial protocols have been followed. Protocol deviations were recorded by the coordinating centre and additional training was carried out if necessary.

### Relevant concomitant care permitted or prohibited during the trial {11d}

During the trial, NHSBT standard care has been in place to ensure donor safety. This includes a screening procedure to ensure the donor is healthy and any medication does not impact donor health or the donation process (including study interventions). As noted above, if a donor has declined to receive the trial intervention, they have been offered standard care.

### Provisions for post-trial care {30}

Given its interventions, this trial is considered minimal risk; no specific post-trial care has, therefore, been provided.

### Outcomes {12}

#### Primary outcome

The primary outcome is the number of in-session VVRs with loss of consciousness (i.e. episodes involving loss of consciousness of any duration, with or without additional complications).

#### Secondary outcomes

Secondary outcomes are (i) all in-session VVRs (i.e. with and without loss of consciousness); (ii) all delayed VVRs (i.e. VVRs with and without loss of consciousness after leaving the donation venue); (iii) delayed VVRs with loss of consciousness; and (iv) any in-session non-VVR adverse events or reactions, such as bruising and rebleeds.

All outcomes will be assessed at the end of the trial and will involve comparison of event rates in periods with and without interventions applied (Appendix 1). The data on the primary and secondary outcomes will be complete by design, as the recording of on-session adverse events, including VVRs, is mandatory in the blood service.

### Participant timeline {13}

All donors who have attended a blood donation session from 4^th^ November 2019 to the 3^rd^ November 2022 have participated in the study, unless they opted out. A baseline visit has been defined as the first time a donor donated during the trial period.

### Sample size {14}

The trial sample size has been informed by (i) the need to generate evidence sufficiently compelling to influence regulators and policy-makers; (ii) NHSBT's duty of care to 900,000 blood donors per year, making it vital for the service to evaluate even small changes in VVR rates; (iii) NHSBT’s objective to optimise blood collection procedures, improve donor return rates, and improve donor (and staff) well-being and satisfaction; and (iv) the need for appropriate power to study determinants of VVRs, both singly and in combination.

**Fig. 2:**
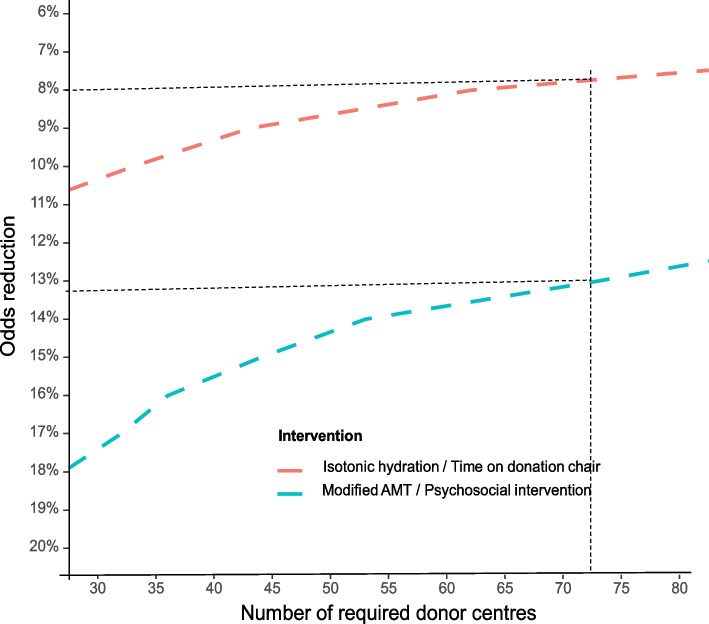
Statistical power of the trial. Calculated for the two interventions in the study were assessed using a cross-over design (i.e. isotonic hydration and time on donation chair after donation; red line) and the two interventions in the study were assessed using a stepped-wedge design (i.e. modified AMT (applied muscle tension) and psychosocial intervention; blue line). Each intervention was tested at a Bonferroni corrected level (type I error) of 0.0125. The study design has four possible sequences for interventions assessed using a stepped-wedge design (X-I-I-I, X-X-I-I, X-X-X-I, X-X-X-X), and two possible sequences for interventions assessed using a cross-over design (X-I-X-I, I-X-I-X) with a study duration of 3 years and each period lasting 9 months (X = current practice, I = intervention). Power calculations assume 15,000 whole blood donations per 9-month period in each donation centre, with an overall rate of severe (syncope) VVRs of 0.18% per donation and a between-centre variance in log odds of 0.06 (estimated from 2014/15 NHSBT data)

Power calculations have been based on the primary endpoint (defined above), assuming a 5% type I error probability and service-wide cluster randomisation (involving 73 teams with ~15,000 whole blood donations per 9-month period per team, and overall primary outcome rate of 0.18% per donation). For the two interventions being assessed using a cross-over design (i.e. isotonic drink and time on donation chair after donation), there is >90% power to detect an odds reduction of >8% (odds ratio of 0.92). For the two interventions being assessed using a stepped-wedge design (i.e. modified AMT and psychosocial intervention), there is >90% power to detect an odds reduction of >13% (odds ratio of 0.87) (Fig. 2). Hence, the study is powered to detect smaller odds reductions than previously reported in the literature but still of importance to blood services. For example, a 10% risk reduction would prevent ~300 severe VVRs per year in NHSBT. All interventions have been tested versus current practice with a 5% overall family-wise error rate control.

### Recruitment {15}

All individuals who have donated blood in one of NHSBT’s blood donation sessions during the trial period have been included in the trial, unless they opted out using the procedure described above.

## Assignment of interventions: allocation

### Sequence generation {16a}

Randomisation of donation sites (“clusters”) to intervention sequences has been undertaken at the trial’s academic coordinating centre, using a computer program written to implement a previously described method for sequential treatment assignment, balancing for known prognostic factors [26]. Specifically, the program implemented a restricted randomisation procedure [27], ensuring that differences in the following key prognostic characteristics at baseline across the assigned intervention groups remained within predefined tolerance limits: historical VVR rates; total number donors bled; and type of site (i.e. static centre or mobile team). Acceptable randomisations were required to have <10% maximal differences in means of continuous characteristics and a maximal difference of 1 in the sum of binary characteristics between any two intervention groups. No stratification was applied in the randomisation. Randomisation simulations were run to generate 100 acceptable trial designs, which required running 59,819 simulations (or 0.17% acceptance proportion). The final allocation was made at a public event, during which two members of the trial’s independent patient and public involvement and engagement (PPIE) panel selected from the list of 100 randomly generated acceptable allocations, drawing a number between zero and nine from two urns corresponding to the numbering of the list of acceptable trial designs. The randomisation procedure and the statistical code used have been reviewed by an external independent expert who confirmed its validity.

### Concealment mechanism {16b}

During the study period, only two people (the trial statistician and study coordinator) were aware of the study randomisation. NHSBT teams (clusters) were informed of the upcoming intervention changes approximately 1 week prior to commencement to allow them to order supplies to deliver the interventions. This process was repeated for each 9-month period.

### Implementation {16c}

The trial statistician at the academic coordinating centre generated the allocation sequence at the beginning of the trial. Every 9 months, when the intervention allocations changed, the trial statistician contacted staff at NHSBT to roll out the next phase of interventions.

## Assignment of interventions: blinding

### Who will be blinded {17a}

As noted above, participants and analysts have not been blinded to the interventions they have received.

### Procedure for unblinding if needed {17b}

Not applicable, this trial is not blinded.

## Data collection and management

### Plans for assessment and collection of outcomes {18a}

Via a Secure File Transfer Protocol (SFTP), NHSBT staff have shared pseudonymised information about donors’ demographic characteristics and adverse donation events (including VVRs) with authorised staff at the academic coordinating centre. All data shared have been strictly pseudonymised (i.e. stripped of personal identifiers). To enhance collection of information on relevant trial outcomes, NHSBT staff have sent donors SMS text messages immediately after donation visits, asking them to report any adverse events by phoning the NHSBT Customer Care line. All adverse events reported by the donors will be assessed by a nurse at the time of reporting and if considered serious, the coordinating centre is informed, along with the study Sponsor and ethics committee.

### Plans to promote participant retention and complete follow-up {18b}

Not applicable; this trial does not involve the need for participant retention or follow-up beyond the mechanisms described above.

### Data management {19}

A formal Data Sharing Agreement was agreed between NHSBT and the University of Cambridge, prior to the start of the STRIDES trial. On a daily basis, NHSBT staff have forwarded to an authorised data manager at the academic coordinating centre pseudonymised extracts from NHSBT’s database including information on donors: donation history, attendance and deferrals; adverse events; microbiology results; merged donor records; and the right to be forgotten. Reports have been in .csv format (accessible only to authorised staff) and uploaded to the SFTP site. These daily extracts have been imported into a password-protected restricted access SAS database where the provided anonymous ID (GUID) has been replaced by a separate anonymous study ID; the link table between GUID and study ID has been available only to an authorised data manager at the academic coordinating centre.

Queries are automatically generated by the STRIDES Data Management systems (written in SAS) by cross-checking/looking for inconsistencies in the data. Resulting queries are resolved by the study helpdesk, by either looking things up in NHSBT systems or contacting the participant directly. Once resolved, any updates/corrections are entered into an Access database by the helpdesk. The Access database is then automatically read into the Data Management systems and updates corrections are automatically implemented. The Data Management Team is responsible to review the data and ensure it is clean prior to analyses.

### Plans to give access to the full protocol, participant-level data and statistical code {31c}

Data access requests should be made to donorhealth@medschl.cam.ac.uk.

### Confidentiality {27}

Not applicable; no personal, identifiable information is collected as part of this trial.

### Plans for collection, laboratory evaluation and storage of biological specimens for genetic or molecular analysis in this trial/future use {33}

During the trial period, a subset of donors have been asked if they wish to join the STRIDES/NIHR BioResource – Research Tissue Bank (henceforth, ‘STRIDES BioResource’), in keeping with the trial’s secondary objective to advance understanding of the determinants of VVRs and to help develop prevention strategies for VVRs tailored to specific donor sub-populations, based on demographic, biological, psychosocial, and other characteristics. Donors who consent to participate in the STRIDES BioResource have been asked to provide a 20-ml blood sample (at the session) and to complete online questionnaires (after the session) concerning VVRs and broader health and donation questions. A full blood count has been performed from the collected blood sample using a Sysmex-XN haematology analyser (Sysmex UK Limited, Milton Keynes, UK) to generate an extended profile of blood cell indices. Full blood count results were reviewed by NHSBT staff to identify clinically significant results for further consideration. As the STRIDES BioResource is part of the NIHR BioResource, consenting donors have also joined the NIHR BioResource Research Tissue Bank (REC: 17/EE/0025) (https://bioresource.nihr.ac.uk/), providing opportunities for participation in additional research projects, subject to provision of further study-specific consent.

## Statistical methods

### Statistical methods for primary and secondary outcomes {20a}

#### Principles

Intention-to-treat analyses will be used. Principal analyses will concern the assessment of the main effects of interventions based on outcomes aggregated at the site level (i.e. unit of randomisation). Tests of interaction will assess whether results differ between pre-specified subgroups. Subsidiary analyses will be done using individual-level data with allowance for clustering of observations by site. Multiple testing will be taken into account when interpreting results other than the principal analyses. The trial will be reported according to CONSORT guidelines.

#### End-of-trial analyses

Primary analyses will calculate odds ratios for the main effects of interventions using a binomial generalised linear mixed model fitted to the aggregate number of primary outcomes recorded in each 9-month period by site with the denominator as the number of donations recorded by site-period (defined by NHSBT as a complete donation or a partial donation). Adjustments will be made for the baseline prognostic variables considered for balancing at randomisation (i.e. historical VVR rates, total numbers of donors bled, and type of site), plus dummy variables for the four nine-month periods of intervention, and a random effect for the site. Key secondary analyses related to the primary outcome will include an assessment of interactions of interventions and possible variation of the main effects by period and baseline characteristics. Analyses of other secondary outcomes will follow the same approach as for the primary outcome.

### Interim analyses {21b}

Not applicable; interim analyses have not taken place as the trial has been judged to be minimal risk.

### Methods for additional analyses (e.g. subgroup analyses) {20b}

Key additional analyses will include an assessment of interactions of interventions and possible variation of the intervention main effects by period and site-level baseline characteristics, including faint rates (tertiles); size as characterised by a number of donors bled (tertiles); and type of site (fixed or mobile). Analyses will follow the same approach as for the primary outcome with the addition of relevant interaction terms. Furthermore, individual-participant data will be secondarily analysed using a hierarchical generalised linear mixed model to assess possible interactions of interventions with selected participant characteristics (e.g. age, sex) accounting for the cluster randomised trial design at the site level.

### Methods in analysis to handle protocol non-adherence and any statistical methods to handle missing data {20c}

As noted above, all analyses will involve the intention to treat principle. Data on outcomes and covariates are expected to be mostly complete as they are anonymously collected from routine blood donation records, with low opt-out rates for use in consented research. We will report on any missing data, but will not conduct data imputation.

## Oversight and monitoring

### Composition of the coordinating centre and trial steering committee {5d}

The Trial Steering Committee monitors the overall conduct of the trial and, through its independent Chair, provides strategic advice to the Trial Management Group. The Trial Steering Committee includes several senior clinical and academic members who are independent from the trial investigators, two lay representatives, and representatives of various stakeholders in STRIDES and meets approximately two to three times per year. Furthermore, the Trial Management Group (which includes the investigators, trial coordinators, members of the PPIE panel, and operational staff from NHSBT) is responsible for overseeing the day-to-day management of the study, liaising with NHSBT, and agreeing protocol amendments prior to submission to the research ethics committee. This Management Group meets monthly throughout the study period.

### Composition of the data monitoring committee, its role and reporting structure {21a}

An independent data monitoring committee was not set up for this study. The main reason for this is the study is minimal risk to human health. The Trial statistician has analysed information on recruitment, baseline characteristics, and total counts of all VVRs recorded approximately two times per year, and presented summary data to the Trial Steering Committee, which then makes the decision on whether the study should continue.

#### Patient and public involvement and engagement

Within the department, there is a well-established panel of public contributors who are current or past blood donors. Two members of the public (blood donors) joined the STRIDES trial management group (TMG) during the design phase and a third member of the public joined six months before the start of the feasibility study. Public members were involved in study design, development of study materials, implementation, governance and decision-making about dissemination of study outcomes. Two additional blood donors sat on two other overarching governance and steering committees. Feedback was also collected from blood donors on the study documentation including patient information leaflet, study consent form, communication text (SMS, website), and design of the interventions (e.g. selecting isotonic drink brand/flavours).

Forms of dissemination will include academic publications, conference presentations, stakeholder meetings, and workshops with blood donors. For the public and other relevant stakeholders, the trial protocol and results will be disseminated via website, email and social media. We will also prepare materials to share key information with NHSBT staff, blood donors and the general public. Lay materials will be co-designed with blood donors and NHSBT, to ensure they are relevant, understandable and impactful.

### Adverse event reporting and harms {22}

Any serious adverse events that occurred during or immediately after the study period will be reported to the Research Ethics Committee and NHSBT by email (cambridgesouth.rec@hra.nhs.uk) within 24 h days, according to the Health Research Authority guidelines. Adverse events that are not serious are recorded and the sponsor of the study is informed within 15 days.

### Frequency and plans for auditing trial conduct {23}

As noted above, during the trial period, each blood donation team within NHSBT has been audited at least once during each intervention period (i.e. every 9 months). During these visits, NHSBT staff members recorded what interventions were in place at the team they visit along with any other relevant observations. The study group then reviewed these findings and identified any potential discrepancies. If any discrepancies appeared, the site has been asked to amend their practices in line with the study protocol.

### Plans for communicating important protocol amendments to relevant parties (e.g. trial participants, ethical committees) {25}

Study amendments, following approval from the Research Ethics Committee and the Sponsor, have been communicated to NHSBT blood donation teams via email. If additional training has been needed, virtual training sessions have been set up to ensure the amendment has been understood and implemented.

### Dissemination plans {31a}

Results from this study will directly inform NHSBT’s blood donation policies. The results will also be published in relevant journals and presented at conferences across the world. Our PPIE members will also be involved in the dissemination of results through our Trial Steering Committee and TMG.

## Discussion

To our knowledge, STRIDES is the largest and most comprehensive cluster randomised trial to date in the prevention of VVR among whole blood donors. The rationale for its conduct was suggested by our prior systematic review and meta-analysis of previous randomised trials assessing the effects of water loading, AMT and other interventions, which highlighted serious limitations in the quality and quantity of available randomised evidence [16].

We anticipate that the STRIDES trial will yield novel information about the four pragmatic and scalable interventions we are assessing, both singly and in combination, for the prevention of VVRs. The trial results should, therefore, help generate policy-shaping evidence to inform blood services to improve donor health, donor experience, and service efficiency.

More generally, this trial exemplifies another instance of NHSBT’s continuing aspiration, in collaboration with strategic academic partners, to be a “learning health organisation” in which internal data and experience are systematically integrated with external evidence, and that knowledge is put into practice to improve safety and efficiency in NHSBT and, potentially, in other blood services [28–32].

## Trial status

Recruitment to the STRIDES study began in November 2019 and will complete in November 2022. Currently protocol version v4.0, 13.07.2020

### Supplementary Information

**Additional file 1:** **Supplementary Figure 1.** The number of blood unitscollected per 10,000 attendances during the feasibility phase of the STRIDEStrial whilst varying combinations of interventions were implemented. **Supplementary Figure 2. **Responses provided by blood donors during the feasibility phase of the STRIDEStrial. **Supplementary Figure 3.** Trialschema. **Supplementary Table 1.** STRIDEStrial randomisation sequence.

## Data Availability

Data access requests should be sent to donorhealth@medschl.cam.ac.uk

## Abbreviations

AMT: Applied muscle tension
CONSORT: Consolidated Standards of Reporting Trials
EU: European Union
NHSBT: National Health Service Blood and Transplant
NIHR: National Institute for Health and Care Research
PPIE: Patient and Public Involvement and Engagement
REC: Research Ethics Committee
SFTP: Secure File Transfer Protocol
SMS: Short Message Service
STRIDES: Strategies to improve donor experiences
TMG: Trial Management Group
VVR: Vasovagal reaction
WHO: World Health Organisation
