# Supplementary material for: Evaluation of interventions to prevent vasovagal reactions among whole blood donors: rationale and design of a large cluster randomised trial
Source: Trials. 2023 Aug 10;24:512. doi: 10.1186/s13063-023-07473-z (PMC10413586; doi:10.1186/s13063-023-07473-z)
Supplement: Supplementary file 1 — Additional file 1: Supplementary Figure 1. The number of blood unitscollected per 10,000 attendances during the feasibility phase of the STRIDEStrial whilst varying combinations of interventions were implemented. Supplementary Figure 2. Responses provided by blood donors during the feasibility phase of the STRIDEStrial. Supplementary Figure 3. Trialschema. Supplementary Table 1. STRIDEStrial randomisation sequence. [file 13063_2023_7473_MOESM1_ESM.docx]

**Supplementary Figure 1:** The number of blood units collected per 10,000 attendances during the feasibility phase of the STRIDES trial whilst varying combinations of interventions were implemented.

| Main effects adjusted for team, month | | | |
| --- | --- | --- | --- |
| Intervention | **parm** | **rr_ci** | **P-value** |
| AMT | 1.amt | 1.01 (0.98, 1.04) | 0.602 |
| PSY | 1.psy | 1.00 (0.98, 1.03) | 0.784 |
| ISO | 1.iso | 1.00 (0.96, 1.03) | 0.803 |
| CHA | 1.cha | 1.02 (0.98, 1.05) | 0.335 |

**Abbreviations:** AMT = New Applied muscle tension; PSY = New Psychosocial handout; ISO = New Isotonic drink; CHA = New Chair time.

**Supplementary Figure 2:** Responses provided by blood donors during the feasibility phase of the STRIDES trial.

**Questions asked:**

New PSY Did you read the new information handout today?

Usual AMT Did you practice the muscle tension exercise at the donation clinic today?

New AMT Did you practice the new muscle tension exercise at the donation clinic today?

Usual H2O How much water did you drink at the donation clinic today prior to donating?

New ISO How much did you drink of the new tablet-based drink at the donation clinic today prior to donating?

**Abbreviations:** AMT = New Applied muscle tension; PSY = New Psychosocial handout; ISO = New Isotonic drink; CHA = New Chair time.

**Supplementary Figure 3:** Trial schema

**
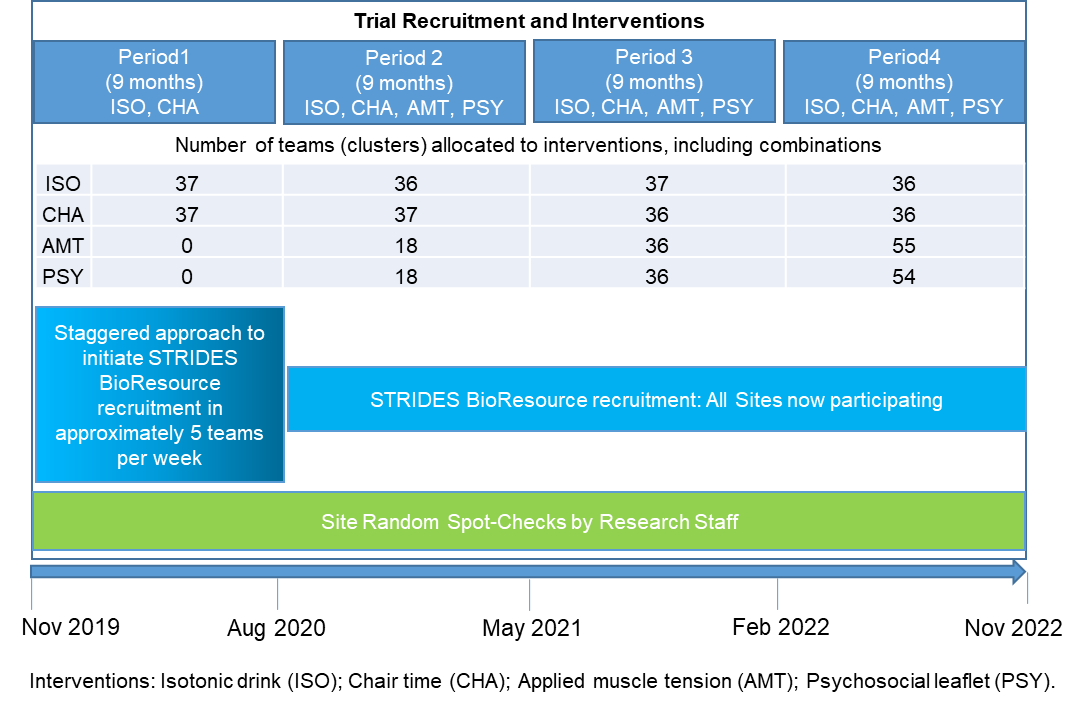
**

**Supplementary Table 1:** STRIDES trial randomisation sequence

73 sites were randomised to possible sequences for conducting interventions during four 9-month periods (Period 1 to Period 4). The sequences for applied muscle tension (AMT) and psychosocial (PSY) interventions followed a stepped wedge design, whereas the sequences for Isotonic drink (ISO) and time on recovery chair (CHA) interventions followed a cross-over design. Their combination gives a hybrid stepped-wedge, cross-over, factorial trial design, in which sites could conduct one or more interventions (or none) at a period. The indicator variables (0/1) denote the interventions allocated to the sites for each period. The column totals summarise the number of sites conducting each intervention during each period. The row totals summarise the number of periods that each site will have carried out each intervention at the trial conclusion.
